# Prevalence, Antimicrobial Susceptibility, and Molecular Characterization of *Escherichia coli* Isolated From Raw Milk in Dairy Herds in Northern China

**DOI:** 10.3389/fmicb.2021.730656

**Published:** 2021-09-24

**Authors:** Huimin Liu, Lu Meng, Lei Dong, Yangdong Zhang, Jiaqi Wang, Nan Zheng

**Affiliations:** ^1^ Laboratory of Quality and Safety Risk Assessment for Dairy Products of Ministry of Agriculture and Rural Affairs, Institute of Animal Sciences, Chinese Academy of Agricultural Sciences, Beijing, China; ^2^ Key Laboratory of Quality & Safety Control for Milk and Dairy Products of Ministry of Agriculture and Rural Affairs, Institute of Animal Sciences, Chinese Academy of Agricultural Sciences, Beijing, China

**Keywords:** *Escherichia coli*, virulence, antimicrobial resistance, raw milk, Northern China

## Abstract

*Escherichia coli* is a common bacterium in the intestines of animals, and it is also the major important cause of toxic mastitis, which is an acute or peracute disease that causes a higher incidence of death and culling of cattle. The purpose of this study was to investigate *E. coli* strains isolated from the raw milk of dairy cattle in Northern China, and the antibacterial susceptibility of these strains and essential virulence genes. From May to September 2015, 195 raw milk samples were collected from 195 dairy farms located in Northern China. Among the samples, 67 (34.4%) samples were positive for *E. coli*. About 67 *E. coli* strains were isolated from these 67 samples. The prevalence of Shiga toxin-producing *E. coli* (STEC), enterotoxigenic *E. coli* (ETEC), enteropathogenic *E. coli* (EPEC), and enteroinvasive *E. coli* (EIEC) were 9, 6, 4.5, and 1.5%, respectively. Among the virulence genes detected, *stx*1 was the most prevalent (6/67, 9%) gene, followed by *eae* (3/67, 4.5%), and *est*B (2/67, 3%). Moreover, the strains exhibited different resistance levels to ampicillin (46.3%), amoxicillin-clavulanic acid (16.4%), trimethoprim-sulfamethoxazole (13.4%), tetracycline (13.4%), cefoxitin (11.9%), chloramphenicol (7.5%), kanamycin (7.5%), streptomycin (6.0%), tobramycin (4.5%), azithromycin (4.5%), and ciprofloxacin (1.5%). All of the *E. coli* isolates were susceptible to gentamicin. The prevalence of β-lactamase-encoding genes was 34.3% in 67 *E. coli* isolates and 45% in 40 β-lactam-resistance *E. coli* isolates. The overall prevalence of *bla*_SHV_, *bla*_TEM_, *bla*_CMY_, and *bla*_CTX-M_ genes were 1.5, 20.9, 10.4, and 1.5%, respectively. Nine non-pathogenic *E. coli* isolates also carried β-lactamase resistance genes, which may transfer to other pathogenic *E. coli* and pose a threat to the farm’s mastitis management projects. Our results showed that most of *E. coli* were multidrug resistant and possessed multiple virulence genes, which may have a huge potential hazard with public health, and antibiotic resistance of *E. coli* was prevalent in dairy herds in Northern China, and ampicillin should be used cautiously for mastitis caused by *E. coli* in Northern China.

## Introduction

*Escherichia coli* was a common inhabitant of the intestine of animals (Tark et al., 2016). During parturition and early lactation period, *E. coli* was found to usually infect mammary gland of cows, which may cause acute and local mastitis (Hinthong et al., 2017). *Escherichia coli* is the main cause of bacterial mastitis in cows. It is usually short-lived, causing the infection that lasts 2–3days. However, *E. coli* has been displayed to cause persistent infections in a few cases (Lippolis et al., 2017). Pathogenic *E. coli* can cause disease in animals and humans due to virious virulence (Ntuli et al., 2016). Based on the epidemiological, clinical, and pathogenic characteristics, *E. coli* is classified into different pathotypes: Shiga toxin-producing *E. coli* (STEC), enteroaggregative *E. coli* (EAEC), enterotoxigenic *E. coli* (ETEC), enteropathogenic *E. coli* (EPEC), and enteroinvasive *E. coli* (EIEC; Rugeles et al., 2010). Numerous outbreaks associated with *E. coli* in milk and other foods have been reported recently (EFSA-ECDC, 2012; EFSA, 2015; Ombarak et al., 2016). For example, STEC can generate two types of Shiga toxins (*stx*1 and *stx*2), and EPEC can produce *bfp* gene, which were involved in pathogenicity of gastrointestinal tract (Hernandes et al., 2009; Douellou et al., 2016). ETEC can express heat-stable *est* genes that can cause severe diarrhea. EAEC can produce *aggR* gene, which were associated with the generation of biofilm (Medeiros et al., 2013). The *ipa*H gene from EIEC can lead to the occurrence of fever, vomiting, and dehydration in infected children. The higher prevalence of *E. coli* is closely associated with hygiene in raw milk (Radostits et al., 2007). Therefore, the study on *E. coli* in raw milk is significant.

*Escherichia coli* is not only with the potential occurrence, but also with the rapid development of antibiotic resistance bacteria (Ntuli et al., 2016). Inappropriate selection and abuse of antibiotics could lead to antibiotic resistance in bacteria (Da Silva and Mendonça, 2012). Moreover, *E. coli* may develop acquired resistance to other antibiotics by carrying various resistance characteristics on mutation, plasmids, or transposons (Gonggrijp et al., 2016). For example, extended-spectrum β-lactamases *E. coli*, resistant to β-lactam antibiotics including third- and fourth-generation cephalosporins, acquires ESBL by mutation or plasmid-mediated horizontal gene transfer (Freitag et al., 2016). Acquired antibiotic resistance also has a transmission potential to humans and other animals (Ruegg et al., 2014). Raw milk can also facilitate the transmission of antibiotic resistance genes to the human gastrointestinal tract, In addition to the presence of pathogenic bacteria. A better understanding on the resistance profile of *E. coli* isolates will improve our understanding of appropriate treatments for pathogen-related management (Tark et al., 2016). Therefore, monitoring the antibiotic resistance of *E. coli* in raw milk may show the trend or specific characteristics of antibiotic resistance and help to better prevent or more effectively treat mastitis on dairy farm.

Antimicrobial resistance and virulence types in *E. coli* have been studied on raw milk of healthy dairy cattle and of bovine mastitis in a variety of countries, including Northern Italy, Romania, Brazil, Egypt, South Korea, and Thailand (Trevisani et al., 2014; Ombarak et al., 2016; Ribeiro et al., 2016; Tark et al., 2016; Hinthong et al., 2017; Tabaran et al., 2017). However, incidence on antibiotic resistance of *E. coli* from raw milk in Northern China were very limited. Continuous monitoring of the antibiotic resistance and virulence type of *E. coli* could be necessary to evaluate *E. coli* risk in raw milk. Therefore, the objective of the work was to investigate the rate of *E. coli* strains isolated from raw milk in Northern China, and to characterize the antimicrobial susceptibility and key virulence genes of these strains.

## Materials and Methods

### Collection of Samples

**Figure 1:**
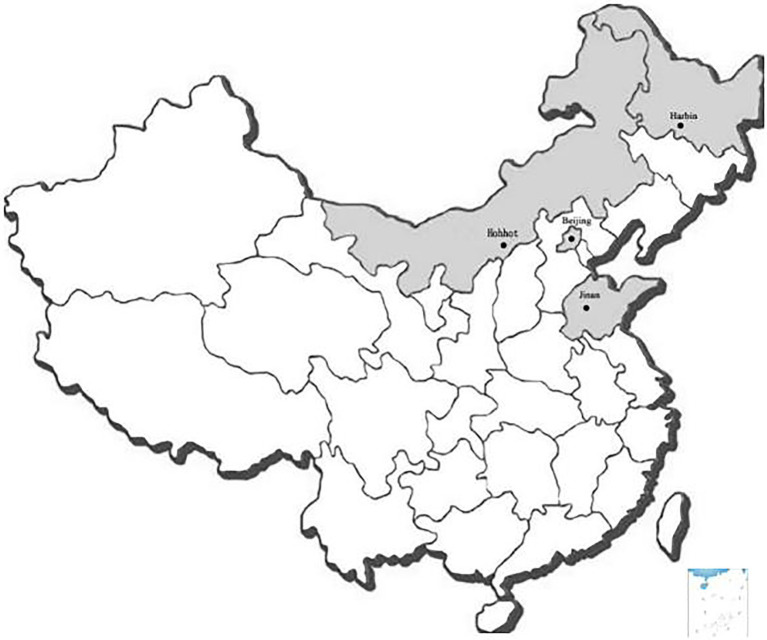
Map of sampling locations. In total, 195 samples were collected from Hohhot, Beijing, Harbin, and Jinan.

In total, 195 raw milk were collected from 195 dairy farms from four cities, which was the major dairy-production cities of Northern China (herd size ≥300, no clinical mastitis cow, milking frequency two or three times per day), from May to September in 2015 (average daily temperature >20°C). There were 30 raw milk samples from Jinan, 40 samples from Harbin, 50 samples from Beijing, and 75 samples from Hohhot (Figure 1). The raw milk samples were collected from the top, middle, and bottom of bulk tank, mixed well, and then transferred into sterile bottles and transported to laboratory at 4°C immediately.

### Isolation and Identification of *E. coli*

Aliquots (25ml) of each sample were added to 225ml tryptic soy broth, and then incubated at 37°C for 16h with shaking for *E. coli* detection. The samples were placed onto Eosin Methylene Blue agar plates (Beijing Land Bridge Technology Ltd., Beijing, China). The agar plates were incubated at 37°C for 18–24h. The presumptive colonies (typical blue-black appearance with a metallic green sheen) were picked. All the colonies were sub-cultured onto nutrient agar slants at 37°C for 16h, and then used for biochemical identification. The colonies initially identified as *E. coli* were examined by Voges-Proskauer negative, methyl-red positive and citrate negative. All isolates were stored at −80°C until use.

All the presumptive colonies were confirmed by PCR on 16S rRNA gene detection (Supplementary Table S1). Genomic DNA was extracted with the InstaGene Matrix DNA extraction kit (Bio-Rad Laboratories), based on the manufacturer’s instruction. PCR were performed with the EmeraldAmp Max PCR Master Mix kit (Takara, Dalian, China) followed the instructions of manufacturer. The primers were synthesized by GeneCreate Biological Engineering Co., Ltd. (Wuhan, China). Briefly, 25μl reactions, which contains 12.5μl of 2×EmeraldAmp Max PCR Master Mix kit, 10pmol of each primer, 1μl of extracted DNA and ultrapure water, were prepared. The amplification conditions were as follows: 94°C for 3min; 30cycles of 94°C for 30s, 55°C for 30s, and 72°C for 1min; and 72°C for 10min for a final extension step. Without genomic DNA as negative control and *E. coli* ATCC 25922 as positive control were included in all the PCR assays.

### Detection of Virulence Determinants

Seven virulence genes for each diarrheagenic *E. coli* were detected by PCR method: *stx*1 and *stx*2 for STEC, *est*A, *est*B, and *elt*B for ETEC, *agg*R for EAEC, *bfp* and *eae* for EPEC, and *ipa*H for EIEC. Amplified products were analyzed by agarose gel electrophoresis, and then visualized by SYBR Safe DNA Stain gel staining. All the primers were shown in Supplementary Table S1.

### Antimicrobial Susceptibility Patterns

Antimicrobial patterns for recovered *E. coli* were determined by agar disk diffusion method (CLSI, 2012). Gentamicin (10μg), tobramycin (10μg), streptomycin (10μg), amoxicillin-clavulanic acid (20/10μg), ampicillin (10μg), ciprofloxacin (5μg), azithromycin (15μg), cefoxitin (30μg), chloramphenicol (30μg), tetracycline (30μg), kanamycin (30μg), and trimethoprim-sulfamethoxazole (1.25/23.75μg) were used as antibiotic agents (Oxoid, Basingstoke, United Kingdom). *Escherichia coli* ATCC 25922 and *Staphylococcus aureus* ATCC 6538 were used as quality controls. The experiment was repeated three times.

### Antimicrobial Resistance Genes

Four β-lactamase resistance related genes (*bla*_CMY_, *bla*_SHV_, *bla*_CTX-M_, and *bla*_TEM_) and two tetracycline genes (*tet*A and *tet*B) were detected by multiplex PCR in *E. coli* strains (Supplementary Table S1). The amplification conditions were as follows: 95°C for 5min, 30cycles of 94°C for 30s, 63°C for 90s, and 72°C for 90s, and 72°C for 7min for a final extension step (Ribeiro et al., 2016). *Escherichia coli* strains ATCC 25922 was used as a positive control in each run.

## Results

### Prevalence of *E. coli*

Out of 195 samples, 67 (34.4%) raw milk samples were positive for *E. coli*. Among these 67 raw milk samples, 67 *E. coli* strains were isolated, including 11 strains (36.7%) of 30 Jinan samples, 23 strains (30.7%) of 75 Hohhot samples, 16 strains (40.0%) of 40 Harbin samples, and 17 strains (34.0%) of 50 Beijing samples.

### Virulence Genes

About 20.9% of the isolates (14/67) harbored more than one virulence gene, as shown in Table 1. The prevalence of EAEC, EIEC, EPEC, ETEC, and STEC was 0, 1.5, 4.5, 6, and 9%. Among the virulence genes detected, *stx*1 was the most prevalent gene (6/67, 9%), followed by *eae* (3/67, 4.5%), *est*B (2/67, 3%), *stx2* (1/67, 1.5%), *est*A (1/67, 1.5%), *elt* (1/67, 1.5%), and *ipa*H (1/67, 1.5%). The *aggR* and *bfp* were not discovered in any *E. coli* strains. Among six STEC isolates, there were three isolates (13.0%) from Hohhot, one isolate (9.1%) from Jinan, one isolate (6.3%) from Harbin, and one isolate (5.9%) from Beijing, respectively. There were two *eae*-positive isolates from Hohhot (8.7%), one *eae*-positive isolates from Jinan (9.1%), and no *eae*-positive isolate from Harbin and Beijing. Moreover, the prevalence of ETEC strains were 9.1% from Jinan, 6.3% from Harbin, 5.9% from Beijing, and 4.3% from Hohhot, respectively. For ETEC-related virulence genes, the prevalence of *est*B, *est*A, and *elt* genes were 3.0% (2/67), 1.5% (1/67), and 1.5% (1/67), and there were one *elt*-positive isolate from Jinan (9.1%), two *est*B-positive isolates from Harbin (6.3%) and Hohhot (4.3%), and one *est*A-positive isolate from Beijing (5.9%). The *ipaH* was detected in only one *E. coli* strain from Harbin.

**Table 1:** Virulence genes in *Escherichia coli* from raw cow milk samples.

Tapes of samples	No. of strains	STEC (%)				ETEC (%)				EPEC (%)			EAEC (%)	EIEC (%)	Total (%)
		*stx*1	*stx2*	*stx*1&*stx2*	Total	*est*A	*est*B	*elt*	Total	*eae*	*bfp*	Total	*agg*R	*ipa*H	
Hohhot	23	2 (8.7)	0 (0)	1 (4.3)	3 (13.0)	0 (0)	1 (4.3)	0 (0)	1 (4.3)	2 (8.7)	0 (0)	2 (8.7)	0 (0)	0 (0)	6 (26.1)
Jinan	11	1 (9.1)	0 (0)	0 (0)	1 (9.1)	0 (0)	0 (0)	1 (9.1)	1 (9.1)	1 (9.1)	0 (0)	1 (9.1)	0 (0)	0 (0)	3 (27.3)
Harbin	16	1 (6.3)	0 (0)	0 (0)	1 (6.3)	0 (0)	1 (6.3)	0 (0)	1 (6.3)	0 (0)	0 (0)	0 (0)	0 (0)	1 (6.3)	3 (18.8)
Beijing	17	1 (5.9)	0 (0)	0 (0)	1 (5.9)	1 (5.9)	0 (0)	0 (0)	1 (5.9)	0 (0)	0 (0)	0 (0)	0 (0)	0 (0)	2 (11.7)
Total	67	5 (7.5)	0 (0)	1 (1.5)	6 (9.0)	1 (1.5)	2 (3.0)	1 (1.5)	4 (6.0)	3 (4.5)	0 (0)	3 (4.5)	0 (0)	1 (1.5)	14 (20.9)

### Antimicrobial Susceptibility Testing

The 67 isolates were exanimated by the disk diffusion method for susceptibility to 12 antibiotics. Antibiotic resistance on *E. coli* was observed to ampicillin (46.3%), amoxicillin-clavulanic acid (16.4%), tetracycline (13.4%), trimethoprim-sulfamethoxazole (13.4%), cefoxitin (11.9%), chloramphenicol (7.5%), kanamycin (7.5%), streptomycin (6.0%), tobramycin (4.5%), azithromycin (4.5%), ciprofloxacin (1.5%), and gentamicin (0; Table 2). Among isolates from Hohhot, the resistant to ampicillin (47.8%) was the most frequently observed, followed by amoxicillin-clavulanic acid (17.4%), tetracycline (13.0%), and sulfamethoxazole-trimethoprim (13.0%), and all investigated strains were sensitive to tobramycin, streptomycin, ciprofloxacin, and gentamicin. Among isolates from Jinan, the resistance to ampicillin and cefoxitin (45.5%) was the most frequently observed, and all investigated strains were sensitive to tobramycin, streptomycin, and gentamicin. Among isolates from Harbin, the resistance to ampicillin (43.8%) was the most frequently observed, followed by amoxicillin-clavulanic acid, tetracycline, sulfamethoxazole-trimethoprim, chloramphenicol, streptomycin, and tobramycin (12.5%), and all investigated strains were sensitive to ciprofloxacin and gentamicin. Among isolates from Beijing, the resistance to ampicillin (47.1%) was the most frequently observed, followed by streptomycin (12.5%), amoxicillin-clavulanic acid (11.8%), tetracycline (11.8%), and sulfamethoxazole-trimethoprim (11.8%), and all the investigated *E. coli* isolates were sensitive to ciprofloxacin, azithromycin, and gentamicin. Moreover, 38 strains (71.6%) were resistant to at least one antibiotic, and 13 isolates (19.4%) were resistant to more than three kinds of antibiotics.

**Table 2:** Antibiotic resistance of strains.

Antibiotics	No. (%) of positive strains				
	Hohhot (*n*=23)	Jinan (*n*=11)	Harbin (*n*=16)	Beijing (*n*=17)	Total (*n*=67)
Ampicillin	11 (47.8)	5 (45.5)	7 (43.8)	8 (47.1)	31 (46.3)
Amoxicillin-clavulanic acid	4 (17.4)	3 (27.3)	2 (12.5)	2 (11.8)	11 (16.4)
Tetracycline	3 (13.0)	2 (18.2)	2 (12.5)	2 (11.8)	9 (13.4)
Sulfamethoxazole-trimethoprim	3 (13.0)	2 (18.2)	2 (12.5)	2 (11.8)	9 (13.4)
Cefoxitin	1 (4.3)	5 (45.5)	1 (6.3)	1 (5.9)	8 (11.9)
Chloramphenicol	2 (8.7)	0 (0)	2 (12.5)	1 (5.9)	5 (7.5)
Kanamycin	2 (8.7)	1 (9.1)	1 (6.3)	1 (5.9)	5 (7.5)
Streptomycin	0 (0)	0 (0)	2 (12.5)	2 (11.8)	4 (6.0)
Tobramycin	0 (0)	0 (0)	2 (12.5)	1 (5.9)	3 (4.5)
Azithromycin	1 (4.3)	1 (9.1)	1 (6.3)	0 (0)	3 (4.5)
Ciprofloxacin	0 (0)	1 (9.1)	0 (0)	0 (0)	1 (1.5)
Gentamicin	0 (0)	0 (0)	0 (0)	0 (0)	0 (0)

### Screening of Antibiotic Resistance Genes

The β-lactamase-encoding genes results were presented in Table 3. The prevalence of β-lactamase-encoding genes were 34.3% in 67 *E. coli* isolates and 45% in 40 β-lactam resistance *E. coli* isolates. The overall prevalences of *bla*_SHV_, *bla*_TEM_, *bla*_CMY_, and *bla*_CTX-M_ genes among *E. coli* isolates, which was narrow spectrum extended-spectrum β-lactamase-encoding genes, β-lactamase-encoding genes, AmpC, and β-lactamase-encoding genes, were 1.5, 1.5, 10.4, and 20.9%, respectively. In total, 71.4% of the isolates, which possessed the *bla_TEM_* gene, were resistant to ampicillin. Around 57.1% of *bla*_CMY_ positive isolates were resistant to amoxicillin-clavulanic acid. Five (7.5%) isolates possessing *bla*_TEM_ or *bla*_CMY_ did not suggest β-lactamase antibiotic resistance.

**Table 3:** β-lactamase genotypes identified in β-lactam-resistant *E. coli*.

Antibiotic	No. of positive strains	No. of positive strains				None
		*bla*_SHV_	*bla*_TEM_	*bla*_CMY_	*bla*_CTX-M_	
Ampicillin	26	0	8	1	0	17
Amoxicillin-clavulanic acid	4	0	0	2	0	2
Cefoxitin	3	0	0	0	1	2
Ampicillin, amoxicillin-clavulanic acid	2	0	1	0	0	1
Amoxicillin-clavulanic acid, cefoxitin	2	0	1	1	0	0
Ampicillin, amoxicillin-clavulanic acid, and cefoxitin	3	1	1	1	0	0
None	27	0	3	2	0	22
Total	67	1	14	7	1	44

Moreover, the presence of the *tet* genes, which were conferring resistance to tetracycline, were confirmed in seven tetracycline-resistance strains. None of the studied strains possessed *tet*A (Table 4).

**Table 4:** Antimicrobial resistance genes among *E. coli*.

Phenotype		Genotype	
Antimicrobial agents	No. of positive strains	Resistant genes or genetic elements studied	No. of positive strains
Penicillin G	47	*blac*Z	30
Cefoxitin	23	*cfx*A	13
Tobramycin	3	*ant(4')*-Ia	2
Gentamicin	7	*aac*6*'*-*aph*2''	7
Chloramphenicol	4	*fex*A	0
		*cat*A	0
Tetracycline	7	*tet*K	1
		*tet*K+*tet*L	2
		*tet*M	3
		*tet*M+*tet*L	1
		*tet*A	0
Erythromycin	25	*erm*B	3
		*erm*B+*erm*C	3
		*erm*C	4
		*erm*C+*erm*A	1
		*erm*C+*msr*A	2
		*erm*C+*msr*B	1
		*msr*A	1
		*erm*A and/or *msr*B	0
Kanamycin	8	*ant(4')*-Ia	8
Lincomycin	28	*lin*A	5
Oxacillin	16	*mec*A	16
Streptomycin	5	*ant(6)*-Ia	0
Quinupristin-Dalfopristin	2	*vga*A and/or *vga*B	0

## Discussion

In this research, 34.4% (67/195) of samples were positive for *E. coli* in raw milk. These results are significantly lower than that in previous studies. The incidence of *E. coli* in raw milk in India was 81.1% (Bhoomika et al., 2016), 75% in Bangladesh (Islam et al., 2016), 64.5% in Malaysia (Jayarao and Henning, 2001), and 45% in Northern China (Lan et al., 2017). In contrast, a much lower incidence (22.4%) of *E. coli* was discovered in raw milk in Sharkia Governorate (Awadallah et al., 2016). Moreover, our results are comparable with the findings of Ntuli et al. (2016), who reported 36% prevalence rate in bulk milk in South Africa, and Sharma et al. (2015), who reported 35.63% occurrence rate in raw milk in the Jaipur city of Rajasthan. Overall, the results indicated that *E. coli* is a common strain in raw milk collected from dairy herds of Northern China. The high prevalence of *E. coli* in raw milk and dairy products is a cause of concern because it is related to contamination from fecal sources and the consequent risk of enteric pathogenic microorganisms in food (Ombarak et al., 2016).

An important factor of *E. coli* infections is virulence factors. When *E. coli* carried some virulence genes, they could be potentially harmful to public consumers (Hinthong et al., 2017). In the study, 20.9% (14/67) of the tested raw milk possessing more than one virulence gene tested, may carried potentially pathogenic *E. coli*, as shown in Table 3. STEC, cause a life-threatening sequel, such as neurological disorder and hemolytic syndrome or HUS (Kaper et al., 2004), was found to be the most common pathogenic *E. coli* strain in raw milk. It has been reported that the virulence genes of STEC isolates were commonly implicated in many foodborne STEC outbreaks in the world (Beutin and Fach, 2015). In this study, the most common virulence genes in raw milk samples in Northern China were *stx* genes. The result was in agreement with Suojala et al. (2011), who reported the STEC (*stx*-positive isolates) was the most common *E. coli* type of raw milk with subclinical mastitis in Southern Finland, and by Lambertini et al. (2015), who found that the most frequently detected gene in raw milk of the United States northeastern was *stx*1. However, STEC or *stx* factors has been detected in the farms of United States and European at a low prevalence (Jayarao et al., 2006; Pradel et al., 2008; Van Kessel et al., 2011; Claeys et al., 2013; Ombarak et al., 2016).

Enteropathogenic *E. coli* is responsible for diarrhea in both developing and developed countries. As an important foodborne pathogen, EPEC has high isolation rate in retail foods in China (Zhang et al., 2016). EPEC were isolated from many animals, such as cattle, goat, sheep, chicken, gull, and pigeon (Gomez-Aldapa et al., 2016). In the study, three strains were *eae* genes-positive and *bfp* gene-negative, which could be classified as EPEC. Cortés et al. (2005) and Gomez-Aldapa et al. (2016) found that atypical EPEC strains were found in raw milk in Egypt, Saudi Arabia, and Slovakia. However, there is no report on the *eae*-positive *E. coli* strains found in mastitis cows in Iran and Thailand (Ghanbarpour and Oswald, 2010; Hinthong et al., 2017). Moreover, an increasing frequency of *eae*-negative isolates were postulated to have other putative adherence and virulence associated factors (Gomez-Aldapa et al., 2016). ETEC strains are usually transmitted by contaminated food. In the study, EPEC and ETEC strains were isolated from Hohhot and Jinan. EPEC/ETEC hybrid isolates were related to EPEC strain, and appeared to have acquired virulence genes by horizontal gene transfer (Hazen et al., 2017).

In the study, antimicrobial resistance was most frequently observed to ampicillin (46.3%). The susceptibility to amoxicillin can be predicted by antimicrobial resistance to ampicillin (CLSI, 2012). So the tested *E. coli* isolates may showed a high resistance to amoxicillin. Nam et al. (2009) reported that 32.2% *E. coli* strains from mastitis cow were resistant to ampicillin. However, the resistant rates in the study were much higher than those in South Korea from 2012 to 2015 (Tark et al., 2016) and in Northern Colorado (McConnel et al., 2016). Antibiotic susceptibility of *E. coli* was more important on choosing a suitable antibiotic for mastitis (Wang et al., 2016). The information of antibiotic use for dairy in Northern China has been investigated in our previous survey. Ampicillin was commonly used in dairy mastitis therapy (Liu et al., 2017). So, ampicillin is not a suitable treatment for mastitis caused by *E. coli* in Northern China.

In our previous survey, we found that five antibiotics (penicillin, ciprofloxacin, sulfamethoxazole-trimethoprim, streptomycin, and gentamicin) were commonly used in mastitis cow. In the study, most of tested strains showed an obvious antimicrobial resistance to ciprofloxacin, sulfamethoxazole-trimethoprim, and streptomycin. These results also indicated that there was a correlation between antibiotic use and antimicrobial resistance.

In the study, there were four β-lactamase resistance genes detected. The β-lactamase-encoding genes prevalence was 34.3% in 67 *E. coli* isolates. β-lactamase resistance genes, such as *bla*_CMY_, *bla*_SHV_, *bla*_CTX-M_, and *bla*_TEM_ were detected in nine non-pathogenic *E. coli* isolates. So non-pathogenic *E. coli* can serve as an antibiotic resistance reservoir and could possibly transfer genes to other pathogenic *E. coli* strains, which can pose a threat to mastitis management programs of farm (Hu et al., 2016). The rate of *bla*_CTX-M,_ *bla*_CMY_, *bla*_TEM_, and *bla*_SHV_ genes among *E. coli* was 1.5, 1.5, 10.4, and 20.9% in the study, respectively. The *bla*_TEM_ and *bla*_CMY_ genes were the most common, which is similar to several previous studies (Navajas-Benito et al., 2016; Gomi et al., 2017; Hinthong et al., 2017). The cephalosporins treatment in mastitis cattle also raised the proportion of *bla*_TEM_ in milk samples at the period of withdrawal (*p*<0.05; Dong et al., 2021). The *bla*_CTX-M_, which was the most important ESBL-related gene, it was associated with the geographic area (Su et al., 2016). However, *bla*_CTX-M_ was the most popular gene in Japan, United Kingdom, France, Netherlands, and Germany (Dahmen et al., 2013; Ohnishi et al., 2013; Timofte et al., 2014; Freitag et al., 2016; Santman-Berends et al., 2016).

Around 11.8% of *E. coli* stains showed resistance to tetracycline in the study. However, Su et al. (2016) reported that the tetracycline-resistance prevalence was 51%. Navajas-Benito et al. (2016) reported that antimicrobial resistance for tetracycline was detected in 19.2% of *E. coli* strains, which recovered from air and its surroundings in Spain. Antimicrobial resistance genes to tetracycline were tested in all the tetracycline-resistant isolates, and three tetracycline-resistant isolates harbored one tetracycline resistance gene *tet*B, which was the most frequent gene, and the studied *E. coli* did not possess *tet*A. However, Gomi et al. (2017) found that the prevalent of *tet*A was more than *tet*B in *E. coli* isolates. It was reported that one representative *E. coli* strain (No. JXLQYF114666) contained nine ARGs including *aph*(3'')-Ib, *bla*_TEM-1B_, *bla*_CMY-2_, *aph*(6)-Id, *mdf*A, *sul*2, *tet*B, *cat*A2, and *dfrA14*, which result in resistance to seven important antibiotics classes (Liu et al., 2020). Moreover, the phenotype-genotype discrepancies on the tetracycline-resistant *E. coli* were observed in the study. However, resistance genotypes on tetracycline, gentamicin, kanamycin, and oxacillin correlated well with resistance phenotypes in *E. coli* and *S. aureus* (Gomi et al., 2017). Therefore, it was still necessary to fully account of testing phenotypic susceptibility for resistance (Zhao et al., 2015). Further research should be carried out to analyze the genetic characteristics on antibiotic resistance by whole-genome approach, which may explain the phenotype-genotype discrepancies observed for many strains.

## Conclusion

In conclusion, the antibiotic resistance on *E. coli* isolated from raw milk in Northern China was assessed for the first time. Our data indicated that *E. coli* isolates were widely present in raw milk samples in Northern China. A total 20.9% of the tested *E. coli* possessed one or more virulence genes, which showed a potential pathogenicity. *Escherichia coli* strains exhibited different levels of antimicrobial resistance, except gentamicin. Ampicillin should not be a suitable treatment of dairy herds for mastitis by *E. coli* in Northern China. Majority of *E. coli* were multiple-antibiotic resistant and co-carried many virulence genes, and it may pose great potential risk to public health. The possibility of transferring and transmitting resistance genes, between non-pathogenic and pathogenic *E. coli* isolates, should be evaluated in further studies.

## Data Availability Statement

The original contributions presented in the study are included in the article/Supplementary Material, further inquiries can be directed to the corresponding author.

## Author Contributions

HL, LM, and LD designed and performed the research. YZ helped with the data analysis. JW gave advices to the researchers. NZ gave the opinions on the research design. All authors contributed to the article and approved the submitted version.

## Funding

This research was supported by China Agriculture Research System of MOF and MARA, The Agricultural Science and Technology Innovation Program (ASTIP-IAS12), The Scientific Research Project for Major Achievements of The Agricultural Science and Technology Innovation Program (CAAS-ZDXT2019004) and Project of Risk Assessment on Raw Milk (GJFP2019026).

## Conflict of Interest

The authors declare that the research was conducted in the absence of any commercial or financial relationships that could be construed as a potential conflict of interest.

## Publisher’s Note

All claims expressed in this article are solely those of the authors and do not necessarily represent those of their affiliated organizations, or those of the publisher, the editors and the reviewers. Any product that may be evaluated in this article, or claim that may be made by its manufacturer, is not guaranteed or endorsed by the publisher.
