# Corrigendum: Prevalence, antimicrobial susceptibility, and molecular characterization of *Escherichia coli* isolated from raw milk in dairy herds in Northern China

**DOI:** 10.3389/fmicb.2025.1593120

**Published:** 2025-05-23

**Authors:** Huimin Liu, Lu Meng, Lei Dong, Yangdong Zhang, Jiaqi Wang, Nan Zheng

**Affiliations:** ^1^Laboratory of Quality and Safety Risk Assessment for Dairy Products of Ministry of Agriculture and Rural Affairs, Institute of Animal Sciences, Chinese Academy of Agricultural Sciences, Beijing, China; ^2^Key Laboratory of Quality & Safety Control for Milk and Dairy Products of Ministry of Agriculture and Rural Affairs, Institute of Animal Sciences, Chinese Academy of Agricultural Sciences, Beijing, China

**Keywords:** *Escherichia coli*, virulence, antimicrobial resistance, raw milk, Northern China

In the published article, there was an error in **Table 4, page 6** as published.

For this article, this is a redundant table. It has been removed.

The authors apologize for this error and state that this does not change the scientific conclusions of the article in any way. The original article has been updated.

