# Supplementary material for: Prevalence, Antimicrobial Susceptibility, and Molecular Characterization of Escherichia coli Isolated From Raw Milk in Dairy Herds in Northern China
Source: Front Microbiol. 2021 Sep 24;12:730656. doi: 10.3389/fmicb.2021.730656 (PMC8500479; doi:10.3389/fmicb.2021.730656)
Supplement: Supplementary file 1 [file Table_1.DOCX]

Supplementary materials

Table S1 Target genes and primers used in the PCR reactions carried out in this study.

| Target gene | Forward primer sequence (5’-3’) | Reverse primer sequence (5’-3’) | Product size (bp) | Reference |
| --- | --- | --- | --- | --- |
| 16S | GTTAATACCTTTGCTCATTGA | ACCAGGGTATCTAATCCTGTT | 340 | Malinen et al. (2003) |
| *tet*A | GTGAAACCCAACATACCCC | GAAGGCAAGCAGGATGTAG | 888 | Harel et al. (1991) |
| *tet*B | AGTGGAGCGATTACAGAA | CATATGTCCTGGCGTGTCTA | 158 | Harel et al. (1991) |
| *bla*_SHV_ | CGCCGGGTTATTCTTATTTGTCGC | TCTTTCCGATGCCGCCGCCAGTCA | 1016 | Ribeiro et al. (2016) |
| *bla*_TEM_ | TTGCTCACCCAGAAACGCTGGTG | TACGATACGGGAGGGCTTACC | 708 | Ribeiro et al. (2016) |
| *bla*_CMY_ | TGATGCAGGAGCAGGCTATTCC | CTAACGTCATCGGGGATCTGC | 323 | Ribeiro et al. (2016) |
| *bla*_CTX-M_ | ATGTGCAGYACCAGTAARGTKATGGM | TGGGTRAARTARGTSACCAGAAYCAGCGG | 593 | Ribeiro et al. (2016) |
| *stx*1 | TTAGACTTCTCGACTGCAAAG | TGTTGTACGAAATCCCCTCTG | 530 | Woodward et al. (1992) |
| *stx*2 | TTATATCTGCGCCGGGTCTG | AGACGAAGATGGTCAAAACG | 326 | Woodward et al. (1992) |
| *eae* | CCCGAATTCGGCACAAGCATAAGC | CCCGGATCCGTCTCGCCAGTATTCG | 881 | Oswald et al. (2000) |
| *est*A | TCCCCTCTTTTAGTCAGTCAACTG | GCACAGGCAGGATTACAACAAAGT | 163 | Ngeleka et al. (2003) |
| *est*B | GCAATAAGGTTGAGGTGAT | GCCTGCAGTGAGAAATGGAC | 368 | Ngeleka et al. (2003) |
| *elt* | TCTCTATGTGCATACGGAGC | CCATACTGATTGCCGCAAT | 322 | Tamanai-Shacoori et al. (1994) |
| *ipaH* | GGTTCCTTGACCGCCTTTCCGATACCGTC | GCCGGTCAGCCACCCTCTGAGAGTAC | 619 | Sethabutr et al. (1993) |
| *aggR* | GTATACACAAAAGAAGGAAGC | ACAGAATCGTCAGCATCAGC | 254 | Rúgeles et al. (2010) |
